# Restoration of Axon Conduction and Motor Deficits by Therapeutic Treatment with Glatiramer Acetate

**DOI:** 10.1002/jnr.23440

**Published:** 2014-07-03

**Authors:** Spencer Moore, Anna J Khalaj, Rhusheet Patel, JaeHee Yoon, Daniel Ichwan, Liat Hayardeny, Seema K Tiwari-Woodruff

**Affiliations:** 1 Department of Neurology, UCLA School of MedicineLos Angeles, California; 2 Pharmacology Unit, Global Innovative Research and Development, Teva Pharmaceutical IndustriesNetanya, Israel; 3 Brain Research Institute, UCLA School of MedicineLos Angeles, California

**Keywords:** multiple sclerosis (MS), glatiramer acetate, demyelination, inflammation, neurodegeneration, therapeutics, neural repair, motor deficit, experimental autoimmune encephalomyelitis (EAE), rotarod, axon conduction

## Abstract

Glatiramer acetate (GA; Copaxone) is an approved drug for the treatment of multiple sclerosis (MS). The underlying multifactorial anti-inflammatory, neuroprotective effect of GA is in the induction of reactive T cells that release immunomodulatory cytokines and neurotrophic factors at the injury site. These GA-induced cytokines and growth factors may have a direct effect on axon function. Building on previous findings that suggest a neuroprotective effect of GA, we assessed the therapeutic effects of GA on brain and spinal cord pathology and functional correlates using the chronic experimental autoimmune encephalomyelitis (EAE) mouse model of MS. Therapeutic regimens were utilized based on promising prophylactic efficacy. More specifically, C57BL/6 mice were treated with 2 mg/mouse/day GA for 8 days beginning at various time points after EAE post-induction day 15, yielding a thorough, clinically relevant assessment of GA efficacy within the context of severe progressive disease. Therapeutic treatment with GA significantly decreased clinical scores and improved rotorod motor performance in EAE mice. These functional improvements were supported by an increase in myelinated axons and fewer amyloid precursor protein-positive axons in the spinal cords of GA-treated EAE mice. Furthermore, therapeutic GA decreased microglia/macrophage and T cell infiltrates and increased oligodendrocyte numbers in both the spinal cord and corpus callosum of EAE mice. Finally, GA improved callosal axon conduction and nodal protein organization in EAE. Our results demonstrate that therapeutic GA treatment has significant beneficial effects in a chronic mouse model of MS, in which its positive effects on both myelinated and non-myelinated axons results in improved axon function. © 2014 The Authors. Journal of Neuroscience Research Published by Wiley Periodicals, Inc.

Multiple sclerosis (MS) is a chronic inflammatory, demyelinating disease of the central nervous system (CNS) that leads to axon damage and resultant motor, sensory, and cognitive deficits. Although research is ongoing to elucidate the precise cellular steps that lead to this axon damage, evidence suggests that an autoimmune response mediated by the adaptive immune system contributes to the formation of demyelinated plaques in white matter and subsequent axon damage (Vollmer et al., 2002; Weissert, 2013). MS relapse and associated manifestations of dysarthria, ataxia, and tremors may result from accumulated axon damage during demyelinating inflammatory insults (Weissert, 2013). Recent evidence indicates that gray matter pathology in MS patients, which includes demyelination and activated microglia, contributes to cognitive defects (Bo et al., 2003; Morgen et al., 2006; Niepel et al., 2006). Therefore, it is of great therapeutic interest to characterize disease-modifying agents capable of limiting or reversing the axon damage that contributes to disease burden. The murine model of MS experimental autoimmune encephalomyelitis (EAE) recapitulates the inflammatory, demyelinating lesions of white and gray matter in cortex, corpus callosum (CC), and spinal cord observed in MS patients (Mangiardi et al., 2011). Indeed, axon damage and transection in murine EAE correlate strongly with clinical disease severity (Kornek and Lassmann, 2003).

Glatiramer acetate (GA; Copaxone) is an FDA-approved agent for first-line treatment of MS that significantly reduces relapse rates in relapse-remitting (RR) MS patients (Johnson et al., 2001), reduces the risk of developing clinically definite MS in patients with clinically isolated syndrome (Comi et al., 2009), and suppresses EAE in animal models (Arnon and Sela, 2003). GA, a synthetic analogue of myelin basic protein (MBP), is a random polypeptide composed of the amino acids L-glutamic acid, L-lysine, L-alanine, and L-tyrosine, and its mechanism of action in MS may lie in preferential differentiation of T_reg_ and Th2 helper cells (Duda et al., 2000; Weber et al., 2007), a Th1 to Th2 shift, and upregulation of anti-inflammatory cytokines (Schrempf and Ziemssen, 2007). GA does not cross the blood–brain barrier but acts on the CNS by inducing peripheral Th2 cells that migrate to the CNS by binding to major histocompatability complex class II molecules present on MBP-recognizing antigen presenting cells (APC; for review see Scott, 2013). These bind to receptors on GA-reactive T cells, triggering cytokine production (Scott, 2013). Recently, decreased activation of CNS tissue-damaging microglia was demonstrated in GA-treated MS patients (Ratchford et al., 2012). EAE studies have shown that GA modulates the immune response by increasing production of IL-10 (Begum-Haque et al., 2013) and T_reg_ cells and reducing Th17 cells (Aharoni et al., 2010). Indeed, GA administration at early and late EAE time points significantly reduced clinical disease (Aharoni et al., 2005). Other GA EAE studies have demonstrated preserved axonal integrity in the spinal cord (Gilgun-Sherki et al., 2003) and enhanced neurogenesis and neuroprotection in the hippocampus, cerebellum, and subventricular zones (Aharoni et al., 2005). However, less is known about the effects of GA on MS/EAE CC pathology and its functional correlates. It is well known that CC integrity in MS is compromised by demyelinating lesions, diffuse tissue damage, and abnormalities in neural connectivity, making it a useful surrogate marker of clinically significant brain abnormalities (Boroojerdi et al., 1998; Warlop et al., 2008; Ozturk et al., 2010).

In this study, we investigated the functional effects of GA treatment during EAE along with neural and inflammatory substrates in spinal cord and CC. Specifically, we sought to determine whether GA treatment during chronic EAE preserves myelination and structural and functional integrity of CNS axons. We hypothesized that GA-induced improvement in clinical outcomes of motor coordination in EAE mice would have neuronal correlates with increased axon myelination, structural integrity, and conduction. To examine this, we induced chronic EAE in adult C57BL/6 mice expressing enhanced green fluorescent protein on the proteolipid protein promoter (PLP_EGFP) and treated them daily with GA for 8 days at various time points during EAE. We report here that therapeutic (i.e., initiated after the presentation of clinical disease) GA treatment during chronic EAE reduced clinical disease and improved motor performance. Spinal cords of GA-treated EAE animals showed increased myelination and axonal integrity as well as decreased inflammatory infiltration. Electrophysiologically a functional improvement in CC axon conduction correlated with increased myelination, pro-myelinating cellularity, and preserved nodal architecture. In sum, these findings indicate a prominent functional improvement in EAE mice as a result of therapeutic GA treatment consistent with improved axonal integrity and myelination of CNS neurons.

## MATERIALS AND METHODS

### Animals

Breeding pairs of PLP_EGFP mice on the C57BL/6 background were a kind gift from Dr. Wendy Macklin (University of Colorado, Denver; Mallon et al., 2002). Mice were bred in-house at the University of California, Los Angeles (UCLA) animal facility. All procedures were conducted in accordance with the National Institutes of Health and approved by the Animal Care and Use Committee of the Institutional Guide for the Care and Use of Laboratory Animals at UCLA.

### GA

GA consists of acetate salts of synthetic polypeptides containing four amino acids: l-alanine, l-glutamate, l-lysine, and l-tyrosine (Teitelbaum et al., 1971). GA (average molecular weight of 7,700 kDa) was obtained from Teva Pharmaceutical Industries (Petah Tiqva, Israel). GA was administered daily for 8 consecutive days by subcutaneous (s.c.) injection (2 mg/mouse, in phosphate buffered saline [PBS]) therapeutically, beginning after the appearance of clinical disease (EAE post-induction day 15 onward).

### EAE

Active EAE was induced in 8 week-old sex-matched PLP_EGFP C57BL/6 mice (Tiwari-Woodruff et al., 2007; Crawford et al., 2010; Mangiardi et al., 2011). Specifically, active EAE was induced by immunization with 200 μg of myelin oligodendrocyte glycoprotein (MOG; amino acids 35–55) in combination with *Mycobacterium tuberculosis* in complete Freund's adjuvant on post-immunization days 0 and 7. Additionally, mice were injected with pertussis toxin (500 ng/mouse) on days 0 and 2. Normal animals were administered all but MOG. Mice were monitored and scored daily for clinical disease severity according to the standard EAE grading scale: 0, unaffected; 1, tail limpness; 2, failure to right upon an attempt to roll over; 3, partial hind limb paralysis; 4, complete hind limb paralysis; and 5, moribund. Within each treatment group, the mean clinical score was determined daily, thereby yielding the mean clinical score for that treatment group. Mice were followed clinically for up to 40 days after disease induction.

### Rotorod Motor Performance

Motor performance was tested up to two times per week for each mouse by using a rotorod apparatus (Med Associates, St. Albans, VT; Kumar et al., 2013; Moore et al., 2013). Briefly, animals were placed on a rotating horizontal cylinder for a maximum of 200 sec. The amount of time the mouse remained walking on the cylinder without falling was recorded. Each mouse was tested at speeds of 3–30 rpm and given three trials on any given day. The three trials were averaged to report a single value for an individual mouse, and averages were then calculated for all animals within a given treatment group. The first two trial days prior to the start of immunization (day 0) served as training trials.

### Immunohistochemistry

Formalin-fixed coronal brain sections containing midline-crossing CC above lateral ventricles or dorsal hippocampus were examined by immunohistochemistry using various series of cell type-specific antibodies, as previously described (Tiwari-Woodruff et al., 2007). The following antibodies were used to detect axons: anti-neurofilament 200 kDa (NF200; 1:500; Millipore, Bedford, MA; and 1:1;000; Sigma, St. Louis, MO); astrocytes: anti-glial fibrillary acidic protein (GFAP; 1:1,000; Millipore); oligodendrocyte (OL) lineage cells: anti-oligodendrocyte transcription factor 2 (olig2; 1:500; Millipore); mature OLs: anti-CC1 (1:1,000; GeneTex) and PLP_EGFP fluorescence; myelin: anti-MBP (1:1;000, Millipore); T cells: anti-CD3 (1:1,000; Abcam, Cambridge, MA; and Millipore); and microglia/macrophage/monocyte: leukocyte antigen marker anti-CD45 (1:500; PharMingen, La Jolla, CA) and amyloid precursor protein (APP; 1:1,000; Abcam and Millipore). The fluorescently tagged secondary antibody step was performed by labeling with antibodies conjugated to TRITC, FITC, or Cy5 (Vector, Burlingame, CA; Chemicon, Temecula, CA). IgG control experiments were performed for all primary antibodies, and no staining was observed under these conditions. To assess cell numbers, nuclear stain 4′,6-diamidino-2-phenylindole (DAPI; 2 ng/ml; Molecular Probes, Eugene, OR) was added for 15 min post-secondary antibody addition and prior to final washes. Sections were mounted on slides, allowed to dry, and coverslipped in Fluoromont G (Fisher Scientific, Pittsburgh, PA).

### Microscopy and Quantification

Immunostaining was quantified using unbiased stereology. The dorsal column (DC) was delineated (Fig. 3A) using the drawing tool in ImageJ version 1.29 (Windows version of NIH Image; http://rsb.info/nih/gov/ij), and MBP, GFAP, CD3, and CD45 staining intensity was quantified within this region. NF200^+^ and MBP^+^ axons were counted in the ventral column (VC) of thoracic spinal cord (Fig. 3A). All images (RGB) were converted to gray scale, split, and separated by color channel in ImageJ. To avoid experimenter bias, autoadjustment of brightness, contrast, and threshold of staining signal were carried out in NIH software. A grid plug-in (ImageJ) was used for counting points per area of interest. Adenomatus polyposis coli (CC1)^+^ mature OLs, olig2^+^ OL lineage cells, and CD3^+^ T cells within the CC or spinal cord dorsal column were counted manually using ×10 or ×40 magnification images and compared blindly among normal, vehicle-treated EAE, and GA-treated EAE groups. Inflammatory cells were quantified by counting the number of CD45^+^ and CD3^+^ cells with DAPI^+^ nuclei in delineated thoracic spinal cord dorsal column (and/or delineated CC). Myelin (MBP^+^) and astrocytes (GFAP^+^) were calculated as percentage area intensity within the spinal cord dorsal column (Fig. 3) and delineated CC. Spinal cord axonal densities were calculated by counting the number of NF200^+^ cells in a ×40 image of ventral column of thoracic spinal cords, where coherent and similar diameter axons are present. Myelinated axon densities were calculated by counting axons (NF200^+^) with a clear ring of MBP^+^ myelin staining around them. Damaged axons were calculated by counting APP^+^ axons.

### Electrophysiological Recording

Brain slices (400 μm thick) corresponding approximately to plates 40–48 in the atlas of Franklin and Paxinos (2001) were used for electrophysiology recording, as previously described (Crawford et al., 2010). Compound action potential (CAP) recordings were performed as previously described (Crawford et al., 2009a,b). Stimulation used for evoked CAP was constant-current stimulus-isolated square wave pulses.

### Statistical Analysis

Quantification of immunostaining results was similar to that in previous studies (Tiwari-Woodruff et al., 2007; Crawford et al., 2009b). Specifically, we used two sections (about 400 μm apart)/mouse from electrophysiology-recorded brains and two sections/mouse from formalin-perfused brains. Among the n = 8 mice per treatment group, four mice were used for electrophysiology and four were formalin perfused, resulting in a total of 16 sections per treatment group for immunostaining. To quantify electrophysiology results from each treatment group, recordings from two caudal slices of four animals per two experiments resulted in a total of 16 analyzed recordings. Values are expressed as mean ± SEM. Statistical analysis of mean values was carried out using one-way analysis of variance (ANOVA) and Friedman test (for clinical scores only) or Bonferroni's multiple-comparisons post-test. Differences were considered significant at *P* < 0.05. Statistical analyses were performed in MicroCal Origin (Northampton, MA) or Prism 4 (GraphPad Software, La Jolla, CA).

## RESULTS

### Therapeutic Treatment with 2 mg GA for 8 Days, Initiated After Onset of Clinical Disease, Attenuates EAE Clinical Scores

**Figure 1:**
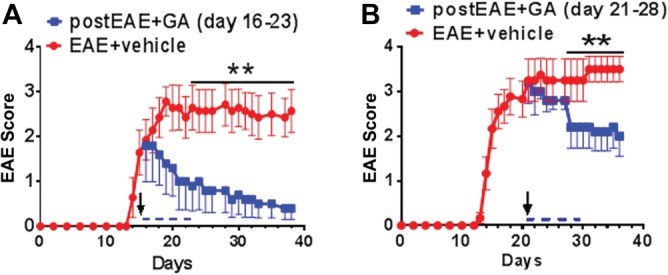
Therapeutic treatment with 2 mg GA for 8 days after onset of clinical EAE attenuates clinical disease scores. PLP_EGFP female mice were administered 2 mg/mouse/day GA (squares) or PBS (vehicle; circles) by s.c. injection on days 16–23 (A) or days 21–28 (B) after initiation of active EAE. Mice were scored using the standard EAE grading scale. EAE scores of mice treated with GA beginning at mid-EAE (day 16) showed significant improvement throughout the duration of disease, whereas EAE mice treated with GA beginning at peak EAE (day 21) showed significant improvement after 8 days of continuous treatment, compared with vehicle-treated EAE mice. Normal mice showed no disease; their clinical scores remained 0 throughout the experiment. Number of mice per treatment group for each experiment: normal, n = 8; EAE + vehicle, n = 8 and 10; post EAE + GA, n = 10 and 15. Data are representative of experiments repeated three times. **P < 0.001, ANOVA, Friedman test. [Color figure can be viewed in the online issue, which is available at http://wileyonlinelibrary.com.]

Given the chronic progressive, inflammatory, and demyelinating nature of MOG_35–55_-induced active EAE in C57BL/6 mice, we hypothesized that a clinically detectable response to GA treatment would be dependent on therapy-initiation timing. To test this hypothesis, we induced active EAE in adult C57BL/6 female mice and tested treatment responses at two time points. Clinical EAE scores were recorded daily by an observer blind to the treatment groups. EAE was clinically evident by postinduction days ∼12–15, accelerated in progression, peaked by days ∼16–21, and subsequently assumed a chronic phase. We identified days 16 (EAE onset) or 21 (peak EAE) as initial time points for an 8 day GA treatment regimen to determine if disease stage causes a differential treatment response and the optimal window of time for therapy. Mice were randomized to receive daily injections of vehicle (PBS) or 2 mg/mouse GA starting on day 16, when the mean EAE score was ∼2 (hindlimb weakness; Fig. 1A), or day 21, when the mean EAE score was ∼3 (partial hindlimb paralysis; Fig. 1B), and followed until day ∼40. Vehicle-treated mice assumed chronic, persistent EAE disability phenotype beginning at day ∼20, as indicated by increased EAE scores. GA treatment initiated at day 16 resulted in suppression of disease phenotype, with treated mice showing a dramatic and persistent reduction in clinical disease burden (Fig. 1A). When GA treatment was initiated at the later time point (day 21), a significant (albeit less dramatic) and persistent decrease in clinical disease burden was observed (Fig. 1B). These results demonstrate the time-dependent efficacy of therapeutic GA treatment, and the persistence of clinical benefit long after cessation of treatment indicates a durable response.

### Therapeutic Treatment with GA Improves Motor Performance in EAE Mice

**Figure 2:**
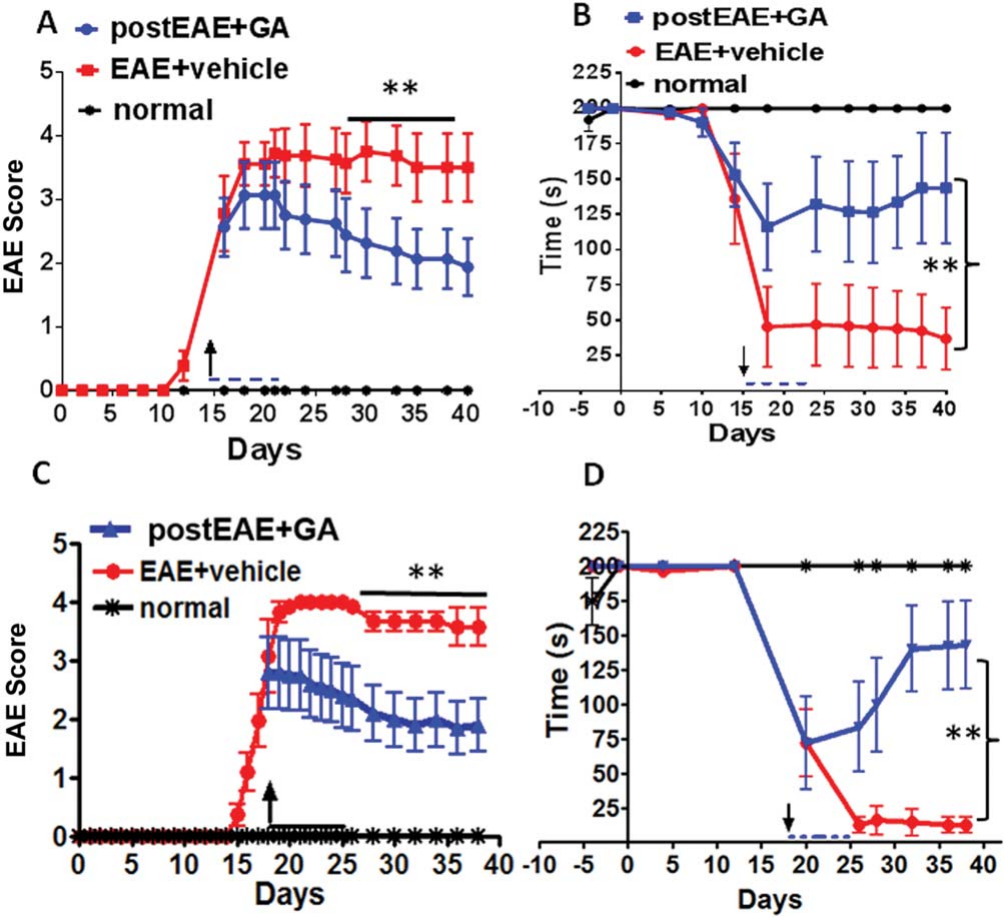
Therapeutic GA decreases EAE-induced motor deficit as measured by rotorod motor performance. PLP_EGFP C57BL/6 female mice were given GA (blue) for 8 days when the average EAE score was ∼3, beginning on EAE post-induction day 15, when the average EAE score was ∼3 (A). In a separate experiment, male mice were given GA (blue) for 8 days when the average EAE score was also ∼3, beginning on day 18 (C). Each experiment contained a group of sex-matched EAE mice administered PBS (vehicle, red). Mice were scored using the standard EAE grading scale. EAE scores of GA-treated mice showed significant improvement throughout the duration of disease compared with vehicle-treated EAE mice. Normal mice (black) did not show disease and their clinical scores remained 0 throughout the experiment. **P < 0.001, ANOVA, Friedman test; n = 10 mice/group. To assess the clinical significance of GA treatment during EAE, mice from experiments in A and C were subjected to a rotorod motor performance test frequently used to assess spinal cord injury. Vehicle-treated EAE mice (red) demonstrated an abrupt and consistent decrease in time (sec) that they were able to remain on the rotorod beginning at day 15 after disease induction, and this disability persisted throughout the observation period. When a group of EAE mice began GA treatment (blue) on day 15 (B) or 18 (D), disability continued but was less severe. However, from days 30–40 the GA-treated EAE group exhibited significant recovery. ** *P* < 0.05, ANOVA; n = 10 mice/group. [Color figure can be viewed in the online issue, which is available at http://wileyonlinelibrary.com.]

To determine whether therapeutic GA-induced improvements in EAE clinical scores were functionally relevant, EAE mice were tested for rotorod motor performance. This behavioral assay of motor coordination and balance in rodents has strong translational correlates with motor assessments of the Kurtzke Expanded Disability Status Scale (EDSS) in MS patients. More specifically, mice are tested for their ability to remain on a rotating cylinder for a maximum of 200 sec per trial, with three trials per day. Although normal, healthy mice remain on the cylinder for the duration of the trial period, clinically severe EAE mice fall off the apparatus much earlier, indicating functional motor disability. Mice were trained on the task prior to EAE induction. Chronic EAE was induced in adult female (Fig. 2A,B) and male (Fig. 2C,D) C57BL/6 mice, as previously described, with a daily 8-day treatment regimen beginning on EAE post-induction day 15 (females) or 18 (males), and mice were sacrificed at day 40 or 36, respectively. For each experiment, GA therapy was initiated when the mean clinical EAE score reached ∼3 (partial hind limb paralysis) to probe the ability of GA both to prevent disease progression and promote motor recovery from severe disease. Mice were randomized to receive either vehicle or GA treatment. EAE clinical scores were obtained daily throughout the experiment by an observer blind to treatment conditions (Fig. 2A,C). Mice were tested on the rotorod several times throughout the disease course. As was observed in earlier experiments, GA treatment significantly suppressed EAE clinical disease in these two experiments, whereas vehicle-treated mice displayed severe and chronic disability beginning at day ∼15 (Fig. 2A,C). Rotorod motor performance declined sharply as EAE clinical disability progressed, but GA treatment rescued performance on the task such that GA-treated mice approached normal performance within 10 days of treatment initiation (Fig. 2B,D). No difference in response to GA treatment, as measured by clinical disease severity and rotorod performance, was observed between males and females. These results reveal that therapeutic GA imparts significant functional motor improvement in clinically severe EAE mice.

**Figure 3:**
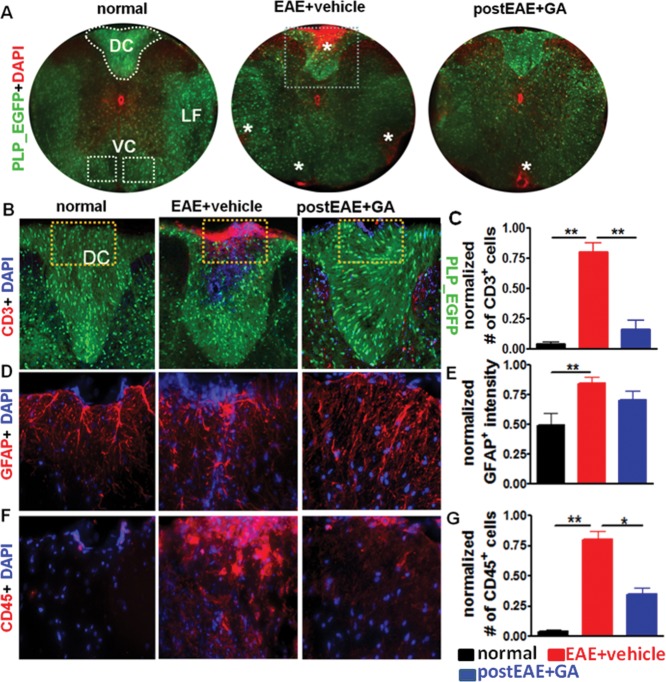
Therapeutic GA reduces inflammation in spinal cords of EAE mice. Representative PLP_EGFP (green) thoracic spinal cord sections costained with DAPI (red) from normal, EAE + vehicle and EAE + GA groups described in Figure 1B, with treatment initiated on peak EAE day 21 (A; ×4 magnification). All animals were sacrificed at EAE post-induction day ∼40. White-dashed perimeters (DC, dorsal column) and boxes (VC, ventral column) denote regions of the spinal cord used for quantification. No inflammatory nuclei are observed in normal controls, whereas vehicle-treated EAE spinal cord shows multifocal to coalescing infiltrates in the leptomeninges. Treatment with 2 mg/day GA for 8 days resulted in fewer DAPI^+^ cell infiltrates in the lateral funiculus (LF), DC, and VC*. Consecutive PLP_EGFP (green) thoracic spinal cord sections co-immunostained for CD3 (B, red, ×10), GFAP (D, red, ×40), or CD45 (F, red, ×40) are shown from partial images (dashed boxes in B) of normal control, vehicle-treated EAE, and GA-treated EAE mice. Vehicle-treated EAE spinal cords had large areas of CD45^+^ and GFAP^+^ cell staining in the DC compared with normal controls, whereas GA-treated EAE mice showed a significant decrease in CD3 and CD45 positivity. Number of CD3^+^ (C), GFAP^+^ (E), and CD45^+^ (G) cells per 400 µm^2^ or cell intensity within the DC were quantified. Compared with normal controls, treatment with GA induced a decrease in CD3^+^ and CD45^+^ cell numbers, but not GFAP^+^ immunoreactivity. Data are representative of experiments repeated in their entirety on another set of EAE mice. *P < 0.05, **P < 0.001, 1 × 4 ANOVA; n = 6–8 mice/group. [Color figure can be viewed in the online issue, which is available at http://wileyonlinelibrary.com.]

### Improvement in Motor Performance with Therapeutic GA Correlates with Reduced Inflammation in Spinal Cords of EAE Mice

Spinal cords of EAE mice reliably show pathological inflammatory lesions and sites of demyelination that contribute to clinical and motor deficits (Mangiardi et al., 2011). To characterize the effects of GA treatment on spinal cord pathology, EAE mice from the Figure 1B experiment, in which treatment was initiated at day 21 (i.e., when demyelination and axonal damage are expected to be more pronounced and challenging to remedy), were sacrificed and formalin-perfused to obtain thoracic spinal cord sections for immunohistochemistry. Inflammation resulting from infiltration by immune cells and astrogliosis are hallmarks of EAE spinal cord pathology. PLP_EGFP^+^ loss and increased DAPI^+^ (which includes inflammatory infiltrates) infiltration observed in the dorsal column of vehicle-treated EAE spinal cord (Fig. 3A, center) were decreased by day 21-initiated GA treatment (Fig. 3A, right). A major infiltrating cell population consisted of CD3^+^ T cells in vehicle-treated EAE spinal cords, as previously observed (Fig. 3B; Mangiardi et al., 2011). T cell numbers, however, were significantly decreased in the spinal cord dorsal column of GA-treated EAE mice (Fig. 3B,C). Activation of resident CD45^+^ CNS microglia is a consistent marker of EAE lesions, and here we report a reduction of CD45^+^ CNS microglia in GA-treated EAE spinal cord dorsal column (Fig. 3F,G). Additionally, astrogliosis is observed in EAE lesions (Voskuhl et al., 2009). We observed GFAP^+^ astrogliosis in both EAE conditions, with a nonsignificant trend toward reduction in the GA-treated group (Fig. 3D,E). Altogether, these results suggest an immunomodulatory effect of GA not on astrocytes but on cells from the hematopoietic line (i.e., T cells and microglia).

### Improvement in Motor Performance with Therapeutic GA Initiated During Late EAE is Associated with Improved Axon Myelination, Improved Axon Numbers, and Reduced Axon Damage

**Figure 4:**
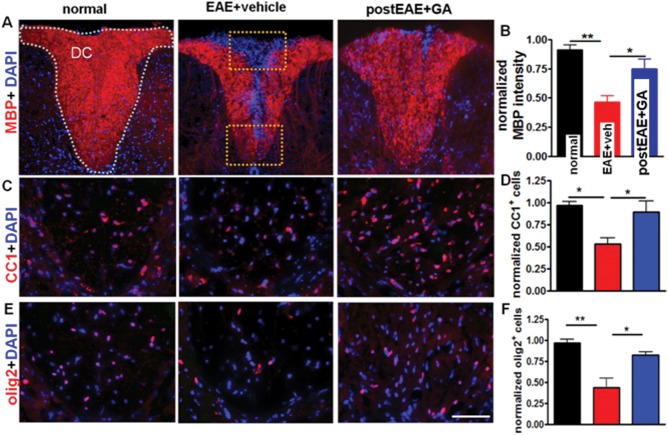
Therapeutic GA restores myelin intensity and OL lineage cells in spinal cords of EAE mice. At EAE post-immunization day 36 (Fig. 1B experiment, in which treatment was initiated on day 21, at peak EAE) vehicle-treated EAE mice had reduced myelin staining intensity (A,B; MBP, red; ×10) and decreased numbers of mature OLs (C,D; APC/CC1, red; ×40) and all OL lineage cells (E,F; Olig2, red; ×40), compared with normal controls, in the DC of thoracic spinal cord sections. In contrast, GA-treated EAE mice showed significant improvement in myelin staining intensity and number of OL cells (A-F). Myelin density and OL numbers are presented as percentage of normal. *P < 0.05, **P < 0.001, 1 × 4 ANOVA; n = 8 mice/group. [Color figure can be viewed in the online issue, which is available at http://wileyonlinelibrary.com.]

Inflammatory demyelination and axon damage are mainly responsible for the axon dysfunction that manifests as clinical and motor deficits in EAE. Axon damage may occur independently or secondarily to demyelination. Thus, the decrease in motor deficits observed in GA-treated EAE mice may be due to a direct or indirect effect of GA on spinal cord axon myelination and axon health. MBP is a reliable marker of CNS myelination. The spinal cord dorsal column, a major myelinated ascending fiber tract, displayed a decrease in MBP staining in vehicle-treated EAE animals (Fig. 4A, center) compared with normal animals. In contrast, the spinal cord dorsal column of GA-treated EAE mice showed improved MBP staining intensity (Fig. 4A, right; Fig. 4B). To explore whether this improvement is attributable to inhibited demyelination and/or accelerated remyelination, we further characterized this myelination effect. Mature myelin-producing OLs were immunostained for CC1. Therapeutic GA induced an increase in CC1^+^ mature OL cell numbers (Fig. 4 C,D). Analysis of all OL lineage cells, including OL progenitors, using OL transcription factor 2 marker olig2 revealed a decrease in olig2^+^ cells in vehicle-treated EAE spinal cord dorsal column and a reversal of this effect in GA-treated EAE spinal cord dorsal column. Taken together, these findings point to reduced loss of OL lineage cells. The myelin-increasing effects of GA are likely to impact axon pathology positively, so they may be functionally relevant.

**Figure 5:**
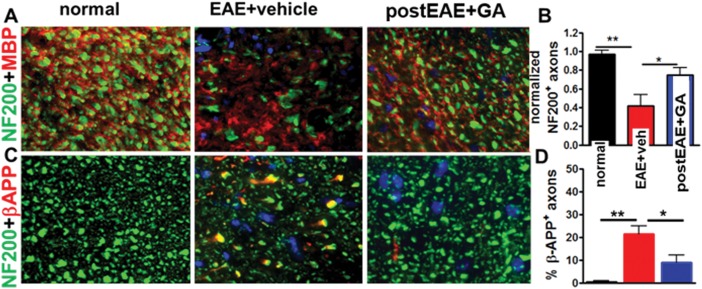
Therapeutic GA reduces axon damage and demyelination in spinal cords of EAE mice. Consecutive thoracic spinal cord sections from normal controls, vehicle-treated EAE, and GA-treated EAE PLP_EGFP mice euthanized on day 36 (Fig. 1B experiment, in which treatment was initiated on day 21 at peak EAE) were immunostained with NF200 (green), MBP (red), and DAPI (blue; A) or NF200 (green), β-APP (red), and DAPI (blue; C). The ventral column (VC; see Fig. 3A, dashed boxes) was imaged at ×40. Axons from the normal group revealed robust NF200 and MBP immunostaining, with the majority of axons showing red myelin around green axons. No APP^+^ axons were visible. In contrast, vehicle-treated EAE sections had decreased myelin and axonal staining, significantly fewer myelinated axons, and significantly more APP^+^ axons. A significant recovery in the number of axons and loss of APP^+^ axons was observed in GA-treated EAE mice. *P < 0.05, **P < 0.001, 1 × 4 ANOVA; n = 6 mice/group (B,D). [Color figure can be viewed in the online issue, which is available at http://wileyonlinelibrary.com.]

To further characterize the effects of GA on axon pathology, we examined the spinal cord ventral column of EAE mice. The pronounced axonal loss (as measured by NF200^+^ stain) observed in vehicle-treated EAE spinal cord was attenuated with GA treatment (Fig. 5A,B). β-APP is known to accumulate in damaged axons (Mangiardi et al., 2011). GA treatment resulted in fewer β-APP^+^ axons compared with vehicle-treated EAE spinal cord ventral column (Fig. 5C,D). Consistent with observed incomplete recovery from clinical signs of EAE, GA treatment partially protected against EAE-induced axonal loss and damage. These findings point to a significant beneficial effect of therapeutic GA in upholding CNS axon numbers and integrity in EAE.

### Therapeutic GA Decreases Lesion Load and Increases PLP_EGFP Fluorescence in EAE Brain

**Figure 6:**
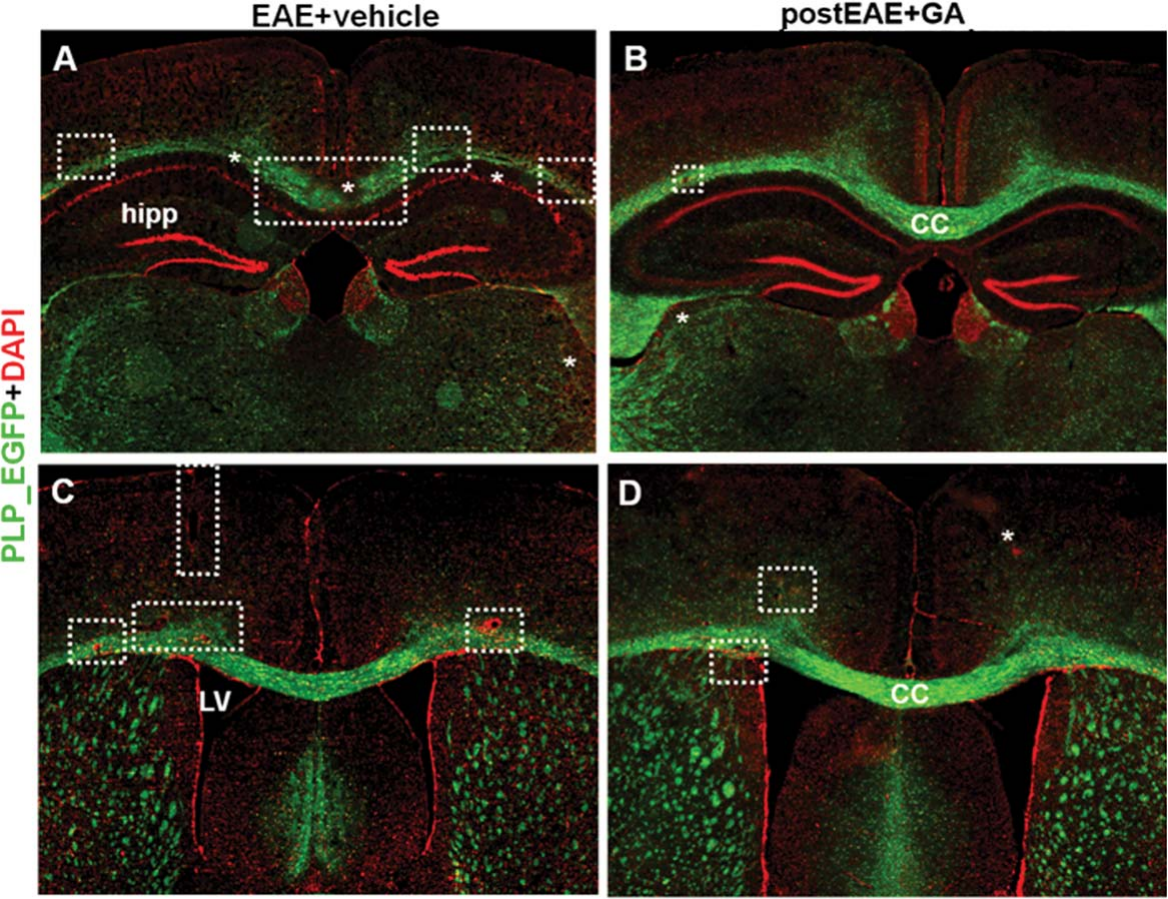
Therapeutic GA increases PLP_EGFP fluorescence in brain sections of EAE mice. Consecutive PLP_EGFP^+^ (green) coronal brain sections corresponding approximately to plates 29–48 in the atlas of Franklin and Paxinos (2001) from the Figure 1B experiment were stained with DAPI (red) and imaged at ×2. Vehicle-treated EAE mice show decreased PLP_EGFP fluorescence, especially in CC and cortical layers, indicated as dashed boxes and asterisks (A). In addition, callosal sections showed many perivascular infiltrating lesions with increased DAPI^+^ nuclei (C, dashed boxes). In contrast, GA-treated EAE mice showed increased PLP_EGFP fluorescence and fewer infiltrating lesions (B,D). [Color figure can be viewed in the online issue, which is available at http://wileyonlinelibrary.com.]

Inflammatory demyelination of white matter tracts of the optic nerve, spinal cord, and brain is a pathological hallmark of MS. Our group has demonstrated consistent inflammatory demyelinated lesions in the CC and cortical gray matter of chronic MOG_35–55_-induced EAE mice (MacKenzie-Graham et al., 2009; Crawford et al., 2010b; Mangiardi et al., 2011; Moore et al., 2013). The CC is the major cerebral commissure and is commonly compromised in MS, in which it frequently displays focal demyelinating lesions and atrophy beginning at early disease stages (Bloom and Hynd, 2005; Audoin et al., 2007). It is therefore important to study how CC damage influences cognitive impairment and physical disability in MS. In addition to assessing GA treatment effects in the spinal cords of EAE animals, we assessed GA effects in the CC of EAE mice. The CC of vehicle-treated PLP_EGFP mice showed reduced PLP_EGFP fluorescence intensity (Fig. 6A,C). This decrease was attenuated with GA treatment (Fig. 6B). Additionally, widespread inflammatory lesions observed in the CC of vehicle-treated EAE mice (Fig. 6B,D; dashed boxes and stars) were reduced with GA treatment. Representative brain sections are from the Figure 1B experiment, in which treatment was initiated at peak EAE (day 21).

### Therapeutic GA Improves Callosal Axon Conduction in EAE Mice

**Figure 7:**
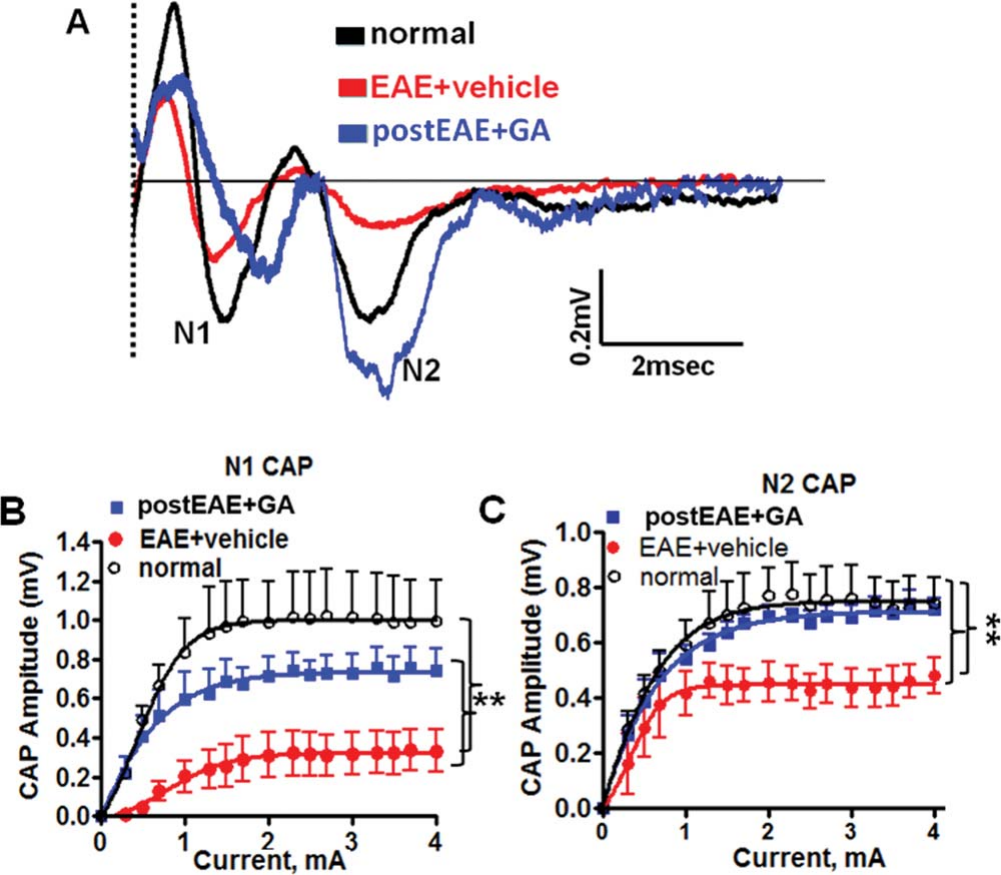
Therapeutic GA mitigates EAE-induced impairment in callosal conduction. A: Callosal lesions and demyelination during chronic EAE cause measurable conduction deficits. CAP responses were recorded on EAE post-immunization day 36 from coronal slices containing midline-crossing segments of the CC overlying the mid-dorsal hippocampus (Fig. 1A experiment). Typical CC CAP from normal (black), vehicle-treated EAE (red), and GA-treated EAE (blue) brains were evoked at a stimulus of 4 mA. N1 (fast conducting, myelinated component) and N2 (slow conducting, mostly non-myelinated component) CAP amplitudes decreased in the vehicle-treated EAE group. Treatment with GA during EAE brought CAP amplitudes closer to those of the normal group by improving the EAE-induced decreases in N1 and N2 CAP amplitudes. Dashed line represents CAP beyond the stimulus artifact. B,C: Quantification of N1 and N2 CAP amplitudes from brain slices of normal, vehicle-treated EAE, and GA-treated EAE groups was performed. CAP amplitudes at 2–4 mA current stimulation were compared. **P < 0.001, ANOVA, Bonferroni's multiple comparison post-test; n = 6 mice/group. [Color figure can be viewed in the online issue, which is available at http://wileyonlinelibrary.com.]

To assess the functional implications of and build upon the mechanistic insight achieved by cellular and structural assessments of GA-induced CNS improvements, we measured callosal axon conduction in coronal slices from vehicle-treated and therapeutic GA-treated EAE animals (subjects from Fig. 1A experiment, in which treatment was initiated on day 16). Our group has routinely studied axon conduction in brain slices by using an electrophysiological assay to measure local field potential change in response to stimulating a CAP in CC with a square wave current pulse (Crawford et al., 2009b, Patel et al., 2013A). With respect to time, the typical CAP shows two distinct voltage deflections: N1, predominantly from large myelinated axons, and N2, predominantly from smaller non-myelinated axons (Fig. 7A). During EAE, both N1 and N2 CAP amplitudes were decreased to nearly 50% of normal (*P* < 0.001; Fig. 7A–C). We report significantly increased N1 and N2 CAP amplitudes in GA-treated EAE CC compared with vehicle-treated EAE CC (*P* < 0.001; Fig. 7A–C). This indicates that GA is neuroprotective of both myelinated and non-myelinated callosal axons. Given our immunohistochemical findings, these functional improvements may be due to a combination of reduced axon loss, increased functional myelination, and reduced axon damage.

### GA-Induced Improvement of Callosal Conduction During EAE is Associated with Decreased Callosal Inflammation and Enhanced Callosal Axon Myelination

**Figure 8:**
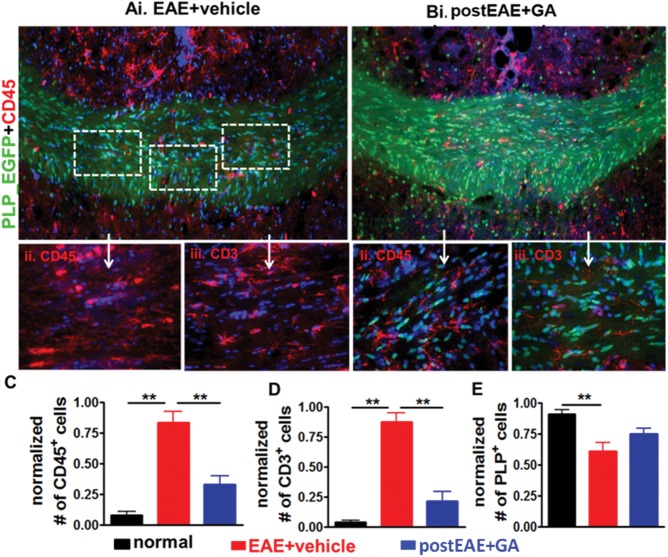
Therapeutic GA decreases EAE-induced callosal inflammation and demyelination. Representative PLP_EGFP brain sections containing CC from vehicle-treated EAE and GA-treated EAE mice (Ai and Bi; Fig. 2A experiment; ×10) were costained with CD45 (red) and DAPI (blue). Dashed boxes denote areas of the spinal cord imaged at ×40 (CD45, A,Bii; CD3, A,Biii). Green channel in Aii and Aiii was removed to show CD45^+^ and CD3^+^ cells clearly. Vehicle-treated EAE spinal cord had multifocal to coalescing infiltrates in the leptomeninges compared with normal controls, where no inflammatory nuclei were observed (Ai). GA treatment during EAE induced a decrease in DAPI^+^ and CD45^+^ cell infiltrates and increased PLP_EGFP fluorescence in the cortical layers and CC (Bi). Numbers of CD45^+^ (C), CD3^+^ (D), and PLP_EGFP^+^ cells (E) per 400 µm^2^ within the delineated CC were quantified. An increase was seen in vehicle-treated EAE mice but not in GA-treated EAE mice, compared with normal controls. Data are representative of experiments repeated in their entirety on another set of EAE mice. **P < 0.001, ANOVA; n = 6–8 mice/group. [Color figure can be viewed in the online issue, which is available at http://wileyonlinelibrary.com.]

Detailed analysis of CC inflammation and myelination was performed in electrophysiologically recorded post-fixed and non-recorded formalin-fixed brain slices from mice in which GA therapy was initiated at EAE day 15 (Fig. 2A). Similar to previous studies (Crawford et al., 2010; Mangiardi et al., 2011; Moore et al., 2013), microglia and T cell-infiltrating lesions in CC of vehicle-treated EAE animals were observed (Fig. 8A,C,D). Activation of resident microglia in the CNS and lymphocytic infiltration into the CNS is pathological and characteristic of EAE (Mangiardi et al., 2011). We report a significant, approximately four-fold reduction of both CD45^+^ activated microglia (Fig. 8B,C) and CD3^+^ T cell infiltrates (Fig. 8B,D) in the CC of GA-treated mice. Whereas the vehicle-treated EAE group displayed a decrease in PLP_EGFP^+^ OLs in delineated CC (Fig. 8A,E), GA-treated EAE CC displayed a non-significant increase in these cells (Fig. 8B,E).

**Figure 9:**
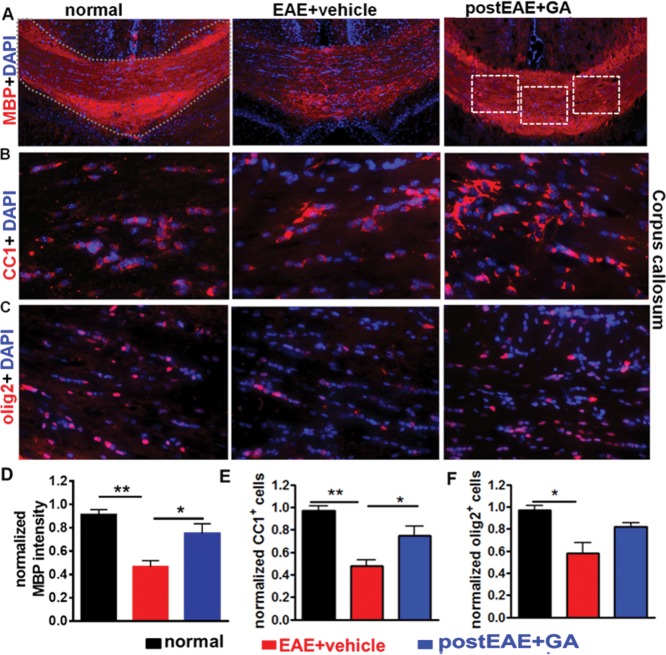
Therapeutic GA during EAE increases myelin density and oligodendrocyte numbers. A,D: Formalin-fixed brain sections from mice euthanized at post-immunization day 40 (Fig. 2A experiment) were immunostained with MBP (red) and DAPI (blue) and imaged at ×10. Vehicle-treated EAE mice had reduced MBP immunoreactivity compared with normal controls. GA-treated EAE mice showed increased MBP staining compared with vehicle-treated EAE mice. Quantification was performed by delineating the CC as shown by dotted lines in the normal panel. Myelin intensity is presented as percentage of normal (*P < 0.05; **P < 0.001 ANOVA, Bonferroni's multiple comparison post-test; n = 8–10 mice/group. B,E: To investigate the potential role of OLs in GA treatment-induced preservation of myelin, brain sections from PLP_EGFP mice were colabeled with DAPI (blue) and mature OL marker APC/CC1 (red) and imaged at ×40 (dashed boxes). Quantification of the number of normalized CC1^+^ cells per 400 µm^2^ indicated a decrease in vehicle-treated EAE mice compared with normal controls. GA treatment caused a significant increase in mature OLs compared with vehicle-treated EAE mice. *P <0.05, **P < 0.001, ANOVA, Bonferroni's multiple-comparisons post-test; n = 8–10 mice/group. C,F: Consecutive sections from normal, vehicle-treated EAE, and GA-treated EAE groups were immunostained with another OL marker (olig2, red) and DAPI (blue) and imaged at ×40. Quantification of the number of normalized olig2^+^ cells per 400 µm^2^ indicated a decrease in vehicle-treated EAE mice compared with normal controls. GA treatment caused a non-significant increase in olig2^+^ cells compared with vehicle-treated EAE mice. *P < 0.05, **P < 0.001, ANOVA, Bonferroni's multiple-comparisons post-test; n = 8–10 mice/group. [Color figure can be viewed in the online issue, which is available at http://wileyonlinelibrary.com.]

MBP staining intensity analysis revealed a significant recovery of callosal myelination in GA-treated EAE mice compared with vehicle-treated EAE mice (Fig. 9A,D). To test the hypothesis that improved myelination effects are attributable to global changes in the OL cell line, we examined OL cell line markers in the CC. CC1^+^ mature OLs were reduced in vehicle-treated EAE CC compared with normal mice. GA increased mature OL numbers in EAE mice (Fig. 9B,E). Similar to results in PLP_EGFP^+^ cells, olig2^+^ OL lineage cells were reduced in vehicle-treated EAE animals, with a non-significant trend toward an increase in GA-treated EAE mice (Fig. 9C,F). Given these results, increased myelination in the CC of therapeutic GA-treated EAE mice may be attributable to enhanced survival and/or differentiation of mature myelin-forming OLs. Overall, these results suggest that therapeutic GA decreases infiltrating cell numbers in the CC, providing an opportunity for recovery from inflammatory demyelination.

### Therapeutic GA Normalizes EAE-Induced Disorganization of Nodal Proteins in Callosal Axons

To assess the extent of GA-induced conduction recovery in callosal axons, nodal architecture was visualized using the paranodal marker Caspr and the nodal sodium channel marker Na_v_1.6. Reorganization of nodal and perinodal axonal constituents precedes remyelination, and Na_v_ channel aggregation is an initial step in the repair process (Coman et al., 2006). Pathological rearrangement of axon ion channels away from typical nodal architecture disrupts normal saltatory action potential conduction, is associated with EAE, and may contribute to clinical deficit (Arroyo et al., 2001; Black et al., 2002; Crawford et al., 2010b). We report significantly fewer Caspr^+^ nodes of Ranvier in the CC of vehicle-treated EAE mice compared with GA-treated EAE mice (from Fig. 2A; Fig. 0A,B). Although nodes of Ranvier are one indicator of axonal integrity, proper saltatory conduction requires colocalization with voltage-gated sodium channels. We observed that the voltage-gated sodium channel Na_v_1.6 colocalized more frequently with Caspr^+^ nodes in the GA-treated condition (Fig. 0A,C). These results suggest that therapeutic GA treatment preserves the proper axon nodal architecture needed for effective callosal conduction and that this change may mediate observed functional improvements in CC axonal conduction.

**Figure:**
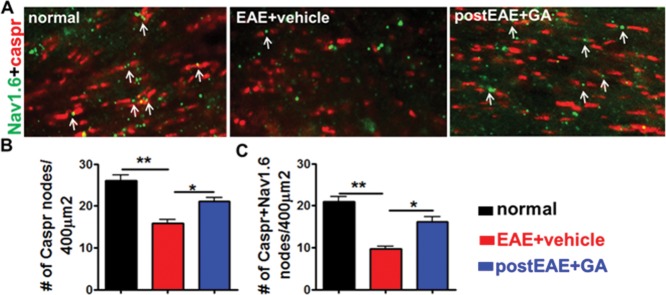
Therapeutic GA treatment limits EAE-induced disorganization of nodal proteins in callosal axons. A: Brain sections (from Fig. 2A experiment) were immunostained with nodal proteins Caspr (red) and Na_v_1.6 (green) and imaged at 100 × magnification. A decrease in Caspr and Na_v_1.6 staining occurred in the CC of vehicle-treated EAE mice. In addition, extensive regions of axons were immunostained, and we found Na_v_1.6 not confined between Caspr pairs. GA-treated EAE callosal axons contained Caspr pairs (arrows) with Na_v_1.6, similar to normal control groups. B,C: Quantification of Caspr protein pairs alone and Caspr protein pair encompassing Na_v_1.6 protein shows a decrease in vehicle-treated EAE callosal axons compared with normal and GA-treated EAE mice. *P < 0.05, **P < 0.001, ANOVA, Bonferroni's multiple comparison post-test; n = 8–10 mice/group. [Color figure can be viewed in the online issue, which is available at http://wileyonlinelibrary.com.]

## DISCUSSION

Recent research has shed light on the immunomodulatory properties that afford GA its disease-modifying efficacy (Aharoni et al., 2010; Kala et al., 2011). However, little was known about the functional outcomes of GA-conferred immunomodulation and neuroprotection in chronic EAE, the most widely accepted animal model of progressive MS. In the current study, we mirrored a typical therapeutic MS treatment paradigm by initiating treatment amidst active chronic clinical disease and studied clinically relevant parameters, including brain pathology and functional correlates. More specifically, we studied the behavioral, physiological, and cellular sequelae of vehicle-treated vs. GA-treated EAE mice to understand how the drug's immunological and neuroprotective properties may modify late disease course. We sought to elucidate the distinct functional efficacy of the drug at different phases of clinically evident disease (i.e., onset to peak), both to increase our understanding of demyelinating disease progression and to optimize therapy. Like chronic and secondary progressive MS, active EAE in C57BL/6 mice is characterized by chronic inflammatory demyelinating lesions in the spinal cord that contribute to sensorimotor deficit, measured with a standard EAE scoring system that resembles the motor components of the Kurtzke EDSS used for MS patients (Mangiardi et al., 2011). In a recent trial studying treatment of RRMS, the most common initial MS subtype, patients who received GA treatment three times per week experienced a reduced risk of EDSS disability progression compared with placebo (Khan et al., 2005). Compared with the discrete EAE clinical scale, in which an experimenter scores the animal's level of weakness or paralysis based on an a priori scale, rotorod testing is a quantitative, more replicable measure of motor function. Its advantages include finer discrimination of motor deficit during early EAE, although it is comparable to the EAE clinical scale for assessing motor deficit in advanced disease. Its use in EAE animals is well-characterized, and latency to fall from the rotorod is inversely correlated with EAE scores (Jones et al., 2008). Here, for the first time, we report significantly improved rotorod motor performance and amelioration of clinical scores in EAE mice treated with therapeutic GA. In addition, we examined pathological markers in the spinal cord to ascertain the mechanism of therapeutic GA-mediated motor improvement during EAE. GA treatment was protective against demyelination in the spinal cord dorsal column. Indeed, reduction in demyelinating inflammatory lesions and preservation of axonal integrity in the spinal cord are major targets of therapy for MS (Mangiardi et al., 2011).

In the present study, GA treatment during mid- to late EAE was associated with reduced inflammatory infiltration and axon damage. These results confirm earlier findings that both prophylactic and therapeutic GA treatment regimens reduce spinal cord pathology and myelin damage in EAE (Aharoni et al., 2008). Increased prevalence of intact axons and reduced loss of spinal cord motor neurons were also observed in GA-treated EAE mice, evidence that GA exerts neuroprotective effects in the spinal cord that mediate clinical improvement (Aharoni et al., 2011). Similar results were found in RRMS patients receiving GA treatment, with reduced spinal cord atrophy compared with interferon-β treatment (Shipova et al., 2009) and reduced microglial activation (Ratchford et al., 2012). In vitro, GA was found to promote brain-derived neurotrophic factor and insulin-like growth factor 1 secretion from microglia (Qian et al., 2013) while increasing the secretion of anti-inflammatory cytokine interleukin (IL)-10 and decreasing the proinflammatory cytokine tumor necrosis factor (TNF)-α (Pul et al., 2011). To further characterize the extent of GA-conferred CNS neuroprotection, we expanded the scope of our study rostrally to the CC.

The CC, one of the largest CNS white matter tracts, is an interhemispheric commissure that subserves cognitive and sensorimotor functions and is subject to demyelinating inflammatory lesions in EAE/MS (Boroojerdi et al., 1998; Mangiardi et al., 2011). Here we report increased OL cellularity and myelination in the CC with therapeutic GA treatment initiated during mid to late EAE. Axonal injury underlies clinical deficit in MS, and topographic redistribution of ion channels away from typical nodal sites may underlie axon dysfunction in the setting of axonal injury. In EAE, the pathologic redistribution of voltage-gated sodium channels 1.2 and 1.6 on the axon was reported by Waxman (2008). We found preserved CC nodal architecture in GA-treated EAE mice with colocalization of sodium channels with nodes of Ranvier, although this arrangement was pathologically disrupted in vehicle-treated EAE mice. The neuroprotective benefits of therapeutic GA treatment were functionally beneficial, inasmuch as we observed increased myelinated and non-myelinated CAP amplitudes in GA-treated EAE mice. Thus, therapeutic GA treatment results in functional improvement that is measurable in vivo.

Our data point to a clear neuroprotective and/or neural repair benefit of GA treatment in EAE, as seen in clinical and physiological manifestations. Although a greater response was seen with GA treatment initiated earlier in disease, a significant suppressive effect on disease progression was observed even during late chronic disease. Here, we show that GA reverses or ameliorates chronic EAE disease burden in mice when treatment is initiated at mid or late disease time points. Specifically, an 8 day GA treatment regimen initiated mid-EAE disease (EAE post-induction day 16) resulted in decreased EAE clinical scores, with animals improving from severe motor disability to only mild tail weakness. When GA treatment was started later in disease (EAE post-induction day 21), animals displayed a significant, albeit less dramatic, reduction in disease burden. These findings demonstrate that GA treatment significantly reduces disease even when administered late in the disease course, and, importantly, the data are indicative of a temporal window within the disease course during which GA treatment is most efficacious. Additionally, the clinical benefits of GA on EAE disease persisted even after the cessation of therapy. Our results mirror those observed in long-term monitoring of GA efficacy in EAE (mice and monkeys; Sela, 1999; Sela and Teitelbaum, 2001; Teitelbaum et al., 2004). Clinical evidence from patients with RRMS supports the neuroprotective effects of GA. GA treatment reduced the proportion of new T1 hypointense lesions transforming to permanent black holes (Filippi et al., 2001). Black holes are indicative of severe tissue damage and may be considered markers of irreversible axonal loss (Filippi et al., 2001). In addition, in another phase III placebo-controlled trial in patients with clinically isolated syndrome, GA treatment was associated with improved neuroaxonal integrity in a subgroup analysis of 34 patients (Comi et al., 2009). A GA treatment trial in progressive MS patients failed to demonstrate a treatment effect (Wolinsky et al., 2007), but a clinincal trial showed that, in MS patients with a mean disease duration of 22 years, administration of GA for up to 15 years reduced relapse rates and decreased disability progression and transition to secondary progressive MS (Ford et al., 2010). Future studies will seek to determine the cellular and molecular nature of the disease stage that promotes response to therapy. Furthermore, additional in vitro and in vivo studies are warranted to determine the precise mechanism by which GA-mediated immunomodulation promotes remyelination and prevents axonal injury. Existing studies point to an immunomodulatory effect of GA on antigen-presenting cells and a shift in T cell populations including Th1 to Th2 (Liblau, 2009). OL biology is another likely target of GA therapy, both directly through growth factors and indirectly through an altered inflammatory environment (Skihar et al., 2009). It is likely that GA promotes a broad cascade of cellular and molecular events across a diverse population of cells that alters the inflammatory milieu of MS/EAE, culminating in disease-modifying effects.
